# Urinary angiotensinogen predicts adverse outcomes among acute kidney injury patients in the intensive care unit

**DOI:** 10.1186/cc12612

**Published:** 2013-04-15

**Authors:** Joseph L Alge, Nithin Karakala, Benjamin A Neely, Michael G Janech, Juan Carlos Q Velez, John M Arthur

**Affiliations:** 1 Department of Medicine, Medical University of South Carolina, 96 Jonathan Lucas St. Charleston, SC, 29425, USA; 2 Medical Service, Ralph H Johnson VA Medical Center, 117 Doughty St. Charleston, SC, 29401, USA

## Abstract

**Introduction:**

Acute kidney injury (AKI) is commonly observed in the intensive care unit (ICU), where it can be caused by a variety of factors. The objective of this study was to evaluate the prognostic value of urinary angiotensinogen, a candidate prognostic AKI biomarker identified in post-cardiac surgery patients, in this heterogeneous population.

**Methods:**

Urinary angiotensinogen was measured by ELISA and corrected for urine creatinine in 45 patients who developed AKI in the ICU. Patients were grouped by AKI etiology, and the angiotensinogen-to-creatinine ratio (uAnCR) was compared among the groups using the Kruskal-Wallis test. The ability of uAnCR to predict the following endpoints was tested using the area under the ROC curve (AUC): the need for renal replacement therapy (RRT) or death, increased length of stay (defined as hospital discharge > 7 days or death ≤ 7 days from sample collection), and worsening AKI (defined as an increase in serum creatinine > 0.3 mg/dL after sample collection or RRT).

**Results:**

uAnCR was significantly elevated in patients who met the composite outcome RRT or death (89.4 vs 25.4 ng/mg; *P *= 0.01), and it was a strong predictor of this outcome (AUC = 0.73). Patients with uAnCR values above the median for the cohort (55.21 ng/mg) had increased length of stay compared to patients with uAnCR ≤ 55.21 ng/mg (22 days vs 7 days after sample collection; *P *= 0.01). uAnCR was predictive of the outcome increased length of stay (AUC = 0.77). uAnCR was also a strong predictor of worsening of AKI (AUC = 0.77). The uAnCR of patients with pre-renal AKI was lower compared to patients with AKI of other causes (median uAnCR 11.3 vs 80.2 ng/mg; *P *= 0.02).

**Conclusions:**

Elevated urinary angiotensinogen is associated with adverse events in AKI patients in the ICU. It could be used to identify high risk patients who would benefit from timely intervention that could improve their outcomes.

## Introduction

Acute kidney injury (AKI) is reflected by an increase in serum creatinine (sCr) or a decrease in urine output, the magnitude of which is used to assess the severity of renal injury using the risk, injury, failure, loss, end-stage renal failure (RIFLE) or Acute Kidney Injury Network (AKIN) staging systems [1,2]. A patient's risk of both short- and long-term adverse outcomes is correlated with the severity of AKI as determined using these staging systems [3-7]. For example, a large retrospective cohort study of a critically ill population reported that the odds ratio (OR) for in-hospital mortality increased from 2.07 in AKIN stage 1 patients to 2.99 in AKIN stage 3 patients [8]. However, because sCr does not reach steady state until after an acute reduction in glomerular filtration rate (GFR) has occurred, the severity of AKI can only be definitively determined late in the disease. The conceptual framework for understanding AKI proposed by Murray *et al*. underscores the importance of progression from the early stages of AKI, in which an at-risk patient experiences renal injury, to later stages of the disease, which include decreased GFR, renal failure, and death [9]. Similarly, the 2012 Kidney Disease: Improving Global Outcomes (KDIGO) Clinical Practice Guideline for AKI highlights the need for accurate assessment of a patient's risk of adverse outcomes, notably progression to a more severe stage of AKI after renal injury has occurred [10].

Unfortunately, it is difficult to determine if a patient with a small increase in sCr will worsen, improve, or stay the same. Furthermore, it is not possible to differentiate mild from severe AKI at an early time point using conventional diagnostic criteria. Biomarkers that reflect the magnitude of tubular injury at the time they are collected could serve this function. Novel AKI biomarkers such as kidney injury molecule 1 (KIM-1), neutrophil gelatinase associated lipocalin (NGAL), IL-18, and Cystatin C can diagnose AKI prior to detectable changes in sCr [11-19]. However, two recent studies have reported unadjusted area under the curve (AUC) values for prediction of worsening AKI between 0.58 and 0.71, suggesting that diagnostic AKI biomarkers are of lesser predictive value among patients who already have established AKI [20,21]. Therefore, prognostic biomarkers that predict outcomes in patients with established AKI are needed.

We recently identified urinary angiotensinogen as a novel prognostic biomarker, capable of predicting adverse outcomes including worsening of AKI and the need for renal replacement therapy after cardiac surgery [22]. However, AKI is a heterogenous syndrome that can be caused by many precipitating factors other than surgery, and it is common among critically ill patients. Because the prognostic predictive power of an AKI biomarker may differ with the pathobiology underlying the etiology it is necessary to determine if the prognostic predictive value of angiotensinogen is generalizable to AKI of other etiologies. Therefore, in the current study we investigated the prognostic predictive power of angiotensinogen in a cohort of critically ill, non-surgical patients in the ICU, who developed AKI.

## Materials and methods

### Patients and urine samples

All patients (*n *= 45) had been admitted to the ICU at the Medical University of South Carolina (MUSC) Hospital. Patients either had AKI at ICU admission or developed AKI during their stay in the ICU. AKI was defined according to the AKIN criteria [2]. When possible, baseline sCr was defined as the most recent (within 1 month) value prior to the AKI episode. When antecedent sCr values were not available, the lowest sCr observed during the patient's hospital stay was used as the baseline. Informed consent was obtained from the patients or their next of kin prior to urine sample collection, in accordance with the MUSC Institutional Review Board approved protocols. The only exclusion criteria were initiation of renal replacement therapy (RRT) prior to sample collection and non-consent. Patients for this study were selected retrospectively to perform a case-control study of ICU patients diagnosed with AKI at the time of urine sample collection. The primary outcome was the need for renal replacement therapy or death, and patients who had AKI at the time of sample collection but did not meet the primary outcome were selected as controls. Decisions about initiating RRT were made by the clinical attending physician. Samples were collected at the time that the diagnosis of AKI was made. If patients had AKI on admission, samples were collected immediately after admission. Urine samples were processed according to a standard operating procedure. They were treated with a protease inhibitor cocktail (Roche, Indianopolis, IN), Mini, ethylenediaminetetraacetic acid (EDTA)-free), centrifuged for 10 minutes at 1,000 × g and the supernatant was aspirated and stored at -80°C until the time of use. Clinical data were obtained by retrospective chart review. Etiology of AKI was determined and patients were assigned to one of four categories: pre-renal, ischemic Acute tubular necrosis (ATN), sepsis-associated AKI, and other. Pre-renal AKI was defined as an episode of AKI in the setting of hypotension or hypovolemia in which the patient's sCr decreased to < 150% of baseline within 48 hours after diagnosis. Ischemic ATN was defined as severe, prolonged AKI following any event that compromises renal blood flow or oxygen delivery. The specific events observed in our cohort included ruptured abdominal aortic aneurysm, cardiogenic shock, and exacerbation of congestive heart failure. Patients for whom the etiology could not be determined or was multifactorial were included in the category, other.

### Determination of urinary angiotensinogen-to-creatinine ratio

Urinary angiotensinogen was measured using the Human Total Angiotensinogen Assay Kit (Immuno-Biological Laboratories Co., Ltd., IBL-America, Minneapolis, MN, USA), a solid-phase sandwich ELISA, according to the manufacturer's protocol. Urine creatinine was measured using the Jaffe assay and used to correct the urine angiotensinogen concentration as was done in the previous analysis of angiotensinogen as a biomarker after cardiac surgery. Values were reported as the ratio of angiotensinogen in ng/ml to creatinine in mg/ml (uAnCR, ng/mg). The AUC value was higher for the primary outcome when creatinine corrected values were used (not shown).

### Outcomes

The primary outcome was the composite outcome of the need for RRT or death. Increased length of hospital stay was defined as hospital discharge > 7 days from the day of sample collection or death ≤ 7 days from sample collection. Worsening of AKI was defined as an additional increase in sCr > 0.3 mg/dL from the sCr at the time of the urine sample collection or the initiation of RRT.

### Statistical analysis

The Kruskal-Wallis test and post hoc Dunn's test were used to compare the uAnCR values of patients grouped by AKI etiology. The Mann-Whitney *U*-test was used when only two groups were compared. Other continuous variables were compared using the *t-*test or Mann-Whitney *U*-test. Categorical variables were compared using the chi squared (χ^2^) or Fisher's exact tests. Logistic regression was used to determine the multiplicative OR for a one SD increase in uAnCR. However, because uAnCR was not normally distributed, it was first log_10 _transformed for this analysis. Receiver operator characteristic (ROC) curves were used to test the ability of uAnCR to predict outcomes, and the AUC was used as an estimate of an overall accuracy of the biomarker. The ROC curve was considered statistically significant if the AUC differed from 0.5, as determined by the *z*-test. Optimal cutoffs were determined by selecting the data point that minimized the geometric distance from 100% sensitivity and 100% specificity on the ROC curve. Additional cutoffs were determined by selecting the points on the ROC at which the positive and negative likelihood ratios were maximized and minimized, respectively. The Spearman's correlation coefficient was used to determine the correlation between uAnCR and length of hospital stay. Kaplan-Meier curves were used to visualize the relationship between uAnCR and length of hospital stay. Patients who died were censored. The log rank test was used to compare the curves. Cox regression was used to calculate the proportional hazard ratio for time to discharge comparing patients with high and low uAnCR (defined as > the median or ≤ the median of the cohort). The Cox proportional hazard model included both the patient's uAnCR and AKIN stage at collection.

## Results

### Patient characteristics

Urine samples were obtained from patients with AKI in the ICU (*n *= 45). At the time of sample collection, five patients were classified as AKIN stage 3, 12 patients as AKIN stage 2, and 28 patients as AKIN stage 1. Baseline patient characteristics are described in detail in Table 1. In approximately one-third of patients, the etiology of AKI could not be determined or was multifactorial (*n *= 16). Sepsis-associated AKI was the most common established etiology (*n *= 15), followed by pre-renal AKI (*n *= 8), and ischemic acute tubular necrosis (*n *= 5). Twenty-three patients met the primary outcome, the need for renal replacement therapy (RRT) or death, of which five patients required RRT but survived, and eleven patients died but did not receive RRT. Pre-renal AKI was significantly more common among the patients who did not meet this outcome (*P *= 0.01). There were no statistically significant differences between the group of patients who required RRT or died compared to those who did not, with respect to age, race, gender, the day of sample collection (defined as days after the date that AKI criteria were met), sCr at baseline, sCr at the time of sample collection, or the percent change in sCr from baseline at the time of sample collection. However, patients who met the primary outcome had lower rates of hypertension, diabetes mellitus, and the use of angiotensin converting enzyme inhibitors or angiotensin receptor blockers.

**Table 1:** Characteristics of ICU patients used to verify the prognostic predictive power of urinary angiotensinogen as an acute kidney injury biomarker

	No RRT and Survival	RRT or Death	*P*-value
Number of patients	22	23	
Age, yrs^a^	62.9 ± 16.1	54.4 ± 17.6	0.1
Caucasian, % (n)	64 (14)	65 (15)	0.84
Male, % (n)	55 (12)	65 (15)	0.67
**AKI etiology, n (%)**			
Sepsis	23 (5)	43 (10)	0.25
Pre-renal	32 (7)	4 (1)	0.01
Ischemic ATN	9 (2)	13 (3)	1
Other	36 (8)	39 (9)	0.91
**Serum creatinine (sCr), mg/dL**			
Baseline sCr^b^	1.15 (0.8, 1.6)	1.1 (1.0, 1.5)	0.98
sCr at collection^a^	2.1 ± 0.8	2.5 ± 0.8	0.06
Change in sCr^b^, %	150 (130 to 189)	200 (150 to 257)	0.07
**AKIN stage at collection**			0.14
Stage 1	17	11	
Stage 2	4	8	
Stage 3	1	4	
**Outcomes, % patients (n)**			
RRT	0	52 (12)	< 0.001
Death	0	78 (18)	< 0.001
**Other variables**			
MAP on day of collection^b^	74.9 (70.4 to 86.8)	68.6 (64.5 to 84.1)	0.08
History of HTN, % patients (n)	91 (20)	48 (11)	0.005
History of diabetes mellitus, % patients (n)	55 (12)	22 (5)	0.05
History of ACE inhibitor or ARB use, % patients (n)	48 (12)	17 (4)	0.03

^a^Mean ± SD; ^b^median and IQR; categorical data are shown as percentage and number. *P*-values are shown for the χ^2 ^or Fisher exact test, as appropriate. RRT, renal replacement therapy; AKI, acute kidney injury; ATN, Acute tubular necrosis}; AKIN, Acute Kidney Injury Network; MAP, Mean arterial pressure}; HTN, Hypertension}; ARB, Angiotensin receptor blocker}.

### Angiotensinogen predicts RRT or death

**Figure 1:**
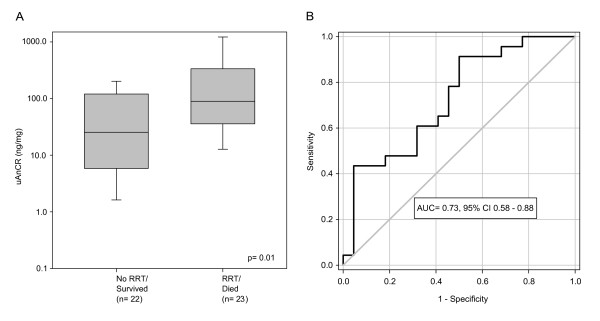
**The urinary angiotensinogen-to-creatinine ratio in patients who met the outcome renal replacement therapy or death compared to patients who did not**. (**A**) Box and whisker plots show the median and interquartile range. Error bars represent the 5^th ^and 95^th ^percentiles. (**B**) A receiver operator characteristic curve was performed to evaluate the ability of the angiotensinogen-to-creatinine ratio (uAnCR) to predict the outcome renal replacement therapy (RRT) or death. The area under the curve (AUC) was 0.73.

Urinary angiotensinogen was elevated in the group of patients who met the primary outcome of RRT or death (median uAnCR = 89.4 ng/mg, IQR 35.9 to 335.6 ng/mg) compared to the group who did not (median uAnCR = 25.4 ng/mg, IQR 5.8 to 120.4 ng/mg) (Figure 1A). Elevated uAnCR was associated with an increased risk of meeting this outcome. The multiplicative OR for a one SD increase in a patient's uAnCR was 2.61 (95% CI 1.23, 5.53). The ROC curve for this outcome had an AUC of 0.73 (*P *= 0.01) (Figure 1B). The optimal cutoff was 34.76 ng/mg, at which the test had a sensitivity and specificity of 78.3% and 54.6%, respectively. The cutoff at which the test had the highest positive likelihood ratio (LR^+ ^= 9.6) was 230.0 ng/mg. Eleven of the forty-five AKI patients had uAnCR values greater than 230.0 ng/mg; ten of these met the outcome. At this cutoff, the sensitivity and specificity of the prediction of RRT or death were 43.5% and 95.5%, respectively, and the positive predictive value was 90.9%. Similarly, the lowest negative likelihood ratio of the test was achieved at a cutoff of 7.58 ng/mg (LR^- ^= 0.14). Eight patients had uAnCR values ≤ 7.58 ng/mg; seven of these did not meet the outcome. The test had a sensitivity and specificity of 95.7% and 31.8%, respectively at this cutoff.

### Length of hospital stay

**Figure 2:**
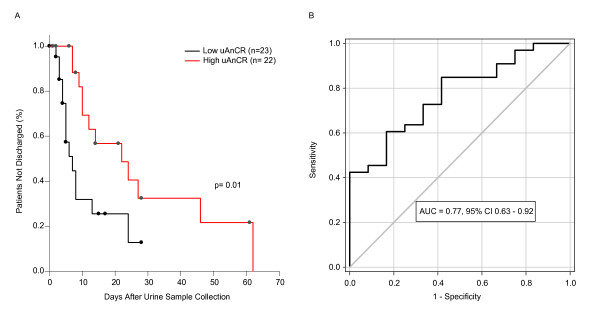
**Urinary angiotensinogen-to-creatinine ratio and length of stay**. (**A**) Patients were stratified into groups by urinary angiotensinogen-to-creatinine ratio (uAnCR). Patients with uAnCR > the median for the cohort were classified as high (red line), whereas patients with uAnCR ≤ the median were classified as low (black line). Patients who died were censored. The median times to discharge (defined as days after sample collection) were 22 and 7 days for the high and low uAnCR groups, respectively. (**B**) Receiver operator characteristic (ROC) curve analysis was performed to evaluate the ability of uAnCR to predict the composite outcome discharge > 7 days after sample collection or death ≤ 7 days from sample collection. AUC, area under the curve.

Among patients who survived to discharge (*n *= 26), uAnCR was correlated with days to hospital discharge (*r *= 0.57, *P *= 0.002). Patients who had high uAnCR values (defined as > 55.21 ng/mg, the median value) had an increased (LOS) compared to patients who had low uAnCR (≤ 55.21 ng/mg). The median LOS (defined as days after the time of sample collection) for these groups was 22 days and 7 days, respectively (*P *= 0.01) (Figure 2A), and the AKIN stage-adjusted hazard ratio for discharge was 0.367 (95% CI 0.17, 0.91) for patients with high uAnCR compared to those with low uAnCR, indicating that uAnCR affects LOS independently of changes in sCr. Elevated uAnCR was strongly associated with an increased risk of the composite outcome discharge > 7 days from the time of sample collection or death ≤ 7 days from collection. The multiplicative OR for one SD increase in uAnCR was 3.31 (95% CI 1.36, 8.04). ROC curve analysis demonstrated that uAnCR was a strong predictor of this outcome (AUC = 0.77) (Figure 2B). At the optimal cutoff, 59.61 ng/mg, the sensitivity and specificity of the prediction of prolonged hospital stay was 60.6% and 83.3%, respectively. The cutoff at which the test had the highest positive likelihood ratio (LR^+ ^= 5.5) was 123.5 ng/mg. Sixteen patients were above this cutoff, of which fifteen met the outcome. At this cutoff, the sensitivity and specificity of the test was 43.5% and 95.5%, respectively. Similarly, the lowest negative likelihood ratio of the test was achieved at a cutoff of 3.31 ng/mg (LR^- ^= 0.12). Four patients had uAnCR values ≤ 3.31 ng/mg; three of these did not meet the outcome. The test had a sensitivity and specificity of 97.1% and 25.0%, respectively at this cutoff.

### Worsening of AKI

**Figure 3:**
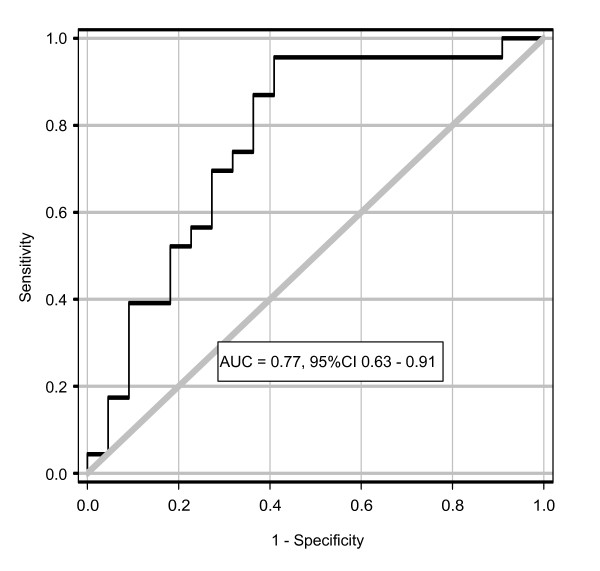
**The urinary angiotensinogen-to-creatinine ratio as a predictor of the outcome worsening of acute kidney injury**. Receiver operator characteristic curve for the composite outcome worsening of acute kidney injury (defined as an increase in serum creatinine > 0.3 mg/dL after the time of sample collection or renal replacement therapy). AUC, area under the curve.

Elevated uAnCR was associated with an increased risk of worsening AKI after sample collection (Figure 3). The ROC curve for this outcome had an AUC of 0.77. At the optimal cutoff, 34.76 ng/mg, the sensitivity and specificity was 87.0% and 63.6%, respectively. At the cutoff with the maximum LR^+^, 230.0 ng/mg (LR^+ ^= 4.31), the sensitivity and specificity was 39.1% and 90.9%, respectively; at the cutoff with the lowest LR^-^, 21.24 ng/mg (LR^- ^= 0.073), the sensitivity and specificity was 95.7% and 59.1%. Eleven patients had uAnCR values above the cutoff of maximal LR^+^; ten of these met the outcome of worsening of AKI. Fourteen patients had uAnCR values below the threshold of minimal LR^-^; only one of these met the outcome.

### Urinary angiotensinogen by AKI etiology

**Figure 4:**
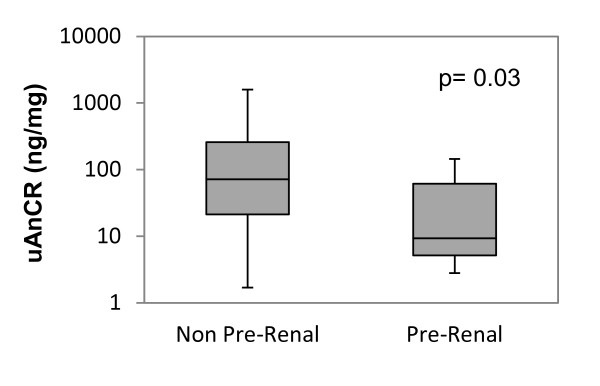
**Urinary angiotensinogen-to-creatinine ratio in pre-renal acute kidney injury (AKI) compared to AKI of other etiologies**. The box and whiskers plots show the median and interquartile range. Error bars represent the 5^th ^and 95^th ^percentiles. Groups were compared with the Mann-Whitney *U*-test. UAnCR, angiotensinogen-to-creatinine ratio.

The uAnCR differed statistically with the underlying etiology of AKI. Patients were categorized into the dichotomous groups of pre-renal AKI and AKI of other etiologies (Figure 4). The median uAnCR for patients with pre-renal AKI (*n *= 8) was 11.3 (IQR 5.2 to 61.5), while the median for patients with AKI not classified as pre-renal etiology (*n *= 37) was 80.2 ng/mg (IQR 22.7 to 259.2). There was a statistically significant difference between the uAnCR values of this group compared to the group of patients with pre-renal AKI (*P *= 0.03). The values for the four separate etiologic groups are shown in Additional file 1. Patients with AKI secondary to ischemic ATN had the highest median uAnCR (260.2 ng/mg, IQR 69.6 to 1213.2), followed by patients with AKI due to other or unknown causes, including multifactorial etiology (90.6 ng/mg, IQR 12.1 to 251.5), patients with sepsis-associated AKI (48.1 ng/mg, IQR 23.5 to 222.4), and patients with pre-renal AKI (11.3, IQR 5.2 to 61.5).

## Discussion

Urinary angiotensinogen has been previously identified as a prognostic biomarker of AKI after cardiac surgery [22]. However, at the time of its discovery it was unclear if its prognostic significance was generalizable to AKI secondary to causes other than cardiac surgery. In the present study, we measured urinary angiotensinogen in ICU patients who developed AKI secondary to diverse etiologies. We found that elevated urinary angiotensinogen was associated with an increased risk of RRT or death, longer time to hospital discharge, and worsening of AKI after the time of sample collection, and that angiotensinogen was a strong predictor of these outcomes using ROC curve analysis (Table 2). Urinary angiotensinogen was statistically decreased in patients with pre-renal AKI compared to those with AKI of other etiologies, and the pre-renal AKI was more common in the group of patients who did not die or require RRT. It is important to distinguish between pre-renal AKI and AKI of other causes since pre-renal AKI typically is transient and resolves with fluid resuscitation, whereas other more severe forms do not. Pre-renal AKI is classically differentiated from AKI of other etiologies by fractional excretion of sodium (FeNa) < 1% or fractional excretion of urea (FeUrea) < 35% [23-25]. However, FeNa can be confounded by diuretic use and is altered in the setting of sepsis, whereas FeUrea decreases with age, and a multicenter trial reported that it was not diagnostic of transient AKI [24-26]. Better biomarkers of pre-renal AKI are clearly needed. In a recent study that used a definition of pre-renal azotemia, which was very similar to ours, cystatin C, NGAL, IL-18 and KIM-1 were elevated in ICU patients with pre-renal AKI compared to those without AKI, but were lower than values for patients whose AKI did not resolve within 48 hrs [27]. An important limitation of our study is that it was a relatively small retrospective biomarker qualification study with 45 subjects. Larger studies will be needed to confirm the prognostic ability of angiotensinogen in this population.

**Table 2:** Summary of performance characteristics of urinary angiotensinogen as a predictor of outcomes among acute kidney injury patients

Outcome	**AUC**^**a**^	Cutoff (ng/mg)	Sensitivity	Specificity
RRT or death^b^	0.73 (0.58 to 0.88)	Best: > 34.76	78.3%	54.6%
		Max LR+: > 230.0	43.5%	95.5%
		Min LR-: ≤ 7.58	95.7%	31.8%

LOS^c^	0.77 (0.63 to 0.92)	Best: > 59.61	60.6%	83.3%
		Max LR+: > 123.5	43.5%	95.5%
		Min LR-: ≤ 3.31	97.1%	25.0%

Worsening AKI^d^	0.77 (0.63 to 0.91)	Best: > 34.76	87.0%	63.6%
		Max LR+: > 230.0	39.1%	90.9%
		Min LR-: ≤ 21.24	95.7%	59.1%

^a^AUC, area under the receiver operator characteristic curve; ^b^RRT, renal replacement therapy; ^c^LOS, length of hospital stay (days after sample collection); ^d^worsening acute kidney injury (AKI) defined as an increase in serum creatinine > 0.3 mg/dL after sample collection or initiation of RRT; LR, likelihood ratio; Max, maximum; Min, minimum.

In this study we also used the maximum LR^+ ^and minimum LR^- ^to define cutoffs at which patients could be stratified into high risk and low risk groups for each outcome, and showed that using these cutoffs, the risk of a significant percentage of the cohort could be assigned with a high level of confidence. We evaluated multiple cutoff values since the purpose of a biomarker affects the threshold that will be used. This approach could be particularly useful for screening patients for enrollment into a clinical trial to enrich the study population with patients who will meet the outcome. For instance, a study to test the efficacy of early initiation of RRT could use as an inclusion criterion a uAnCR value > 230 ng/mg. Using this cutoff we identified 43.5% of the patients who would require RRT or die, while excluding 95.5% of patients who would not meet the endpoint. Therefore, if a target enrollment of 500 is assumed, we would only enroll 45 patients who would not meet the endpoint RRT or death, and who would gain no survival benefit from the intervention. However, using this cutoff we need to screen a total of 2,083 patients to meet the target enrollment. Of the 1,583 patients screened but not enrolled, 589 would eventually meet the endpoint, and so the benefit of enrichment would need to be weighed against the cost of screening.

Our findings could also have important implications for our understanding of the pathobiology of AKI. Angiotensinogen is the principal substrate of renin, and a major driver of activation of the renin-angiotensin system (RAS). Animal studies have suggested a role for the RAS in the molecular mechanisms of AKI. It has been observed that angiotensin II increases and angiotensin 1-7, the molecular counterbalance of angiotensin II decreases in kidney tissue following ischemia reperfusion injury in rats [28,29]. Angiotensin II can contribute to renal injury through pro-inflammatory effects mediated by the nuclear factor-κB (NF-κB) pathway, and it has been demonstrated that inhibition of angiotensin converting enzyme and the angiotensin II type 1 receptor with captopril and losartan, respectively, reduce renal inflammation and mitigate the severity of AKI in rats subjected to renal ischemia reperfusion injury [30,31]. Interestingly, intrarenal angiotensin II concentration strongly correlates with urinary angiotensinogen concentration (*r *= 0.79), but is not correlated with plasma angiotensinogen [32]. Our findings are suggestive of a role of the RAS in modulating the severity of AKI, a notion which is supported by a recent study in which an association was reported between severity of tubular atrophy and urinary angiotensinogen among individuals with chronic kidney disease [33].

## Conclusions

Urinary AnCR could be useful as a prognostic AKI biomarker in the setting of the ICU. It could be used to evaluate a patient's risk of adverse outcomes, potentially leading to an altered interventional strategy or improved enrollment in clinical trials. Angiotensinogen also appears to discriminate between AKI of pre-renal etiology and other etiologies and may be useful to discriminate between patients with pre-renal azotemia and intrinsic renal injury.

## Key messages

• Urinary angiotensinogen could be used as a prognostic biomarker of AKI in the ICU.

• Urinary angiotensinogen predicts adverse outcomes in patients with AKI and could be used in clinical trial design to enrich the study population with patients who might benefit from intervention.

• Urinary angiotensinogen has the potential to be useful in the discrimination of pre-renal AKI from intrinsic AKI.

• Urinary angiotensinogen concentrations could reflect activation of the intrarenal RAS during AKI.

## Abbreviations

AKI: acute kidney injury; AKIN: Acute Kidney Injury Network; AUC; area under the curve of a receiver operator characteristics curve; ELISA: enzyme-linked immunosorbent assay; FENa: fractional excretion of sodium; FEUrea: fractional excretion of urea; GFR: glomerular filtration rate; IL-18: interleukin 18; IQR: interquartile range; KDIGO: Kidney Disease: Improving Global Outcomes; KIM-1: kidney injury molecule 1; LOS: length of stay; LR^+^: positive likelihood ratio; LR^-^: negative likelihood ratio; MUSC: Medical University of South Carolina; NF-kB: nuclear factor -kB; NGAL: neutrophil gelatinase associated lipocalin; OR: odds ratio; RAS: renin angiotensin system; RIFLE: risk, injury, failure, loss, end-stage renal failure; ROC: receiver operator characteristics; RRT: renal replacement therapy; sCr: serum creatinine; uAnCR: urinary angiotensinogen to creatinine ratio.

## Competing interests

The authors have no competing interests.

## Authors' contributions

JLA performed the assays and statistical analysis and wrote the initial draft of the manuscript. NK identified patients for sample collection and participated in the design of the studies. BAN assisted with the assays and statistical analysis. MGJ assisted with the design of studies and analysis. JCQV assisted with design and interpretation of the study. JMA designed the study and assisted with analysis and interpretation of the results. All authors read and approved the final manuscript.

## Supplementary Material

Additional file 1 **Urinary angiotensinogen/creatinine ratio (uAnCR) by acute kidney injury (AKI) etiology**. Patients who developed AKI in the ICU were grouped by the etiology underlying the AKI. The median (black dot) and interquartile range (error bars) are shown. The * symbol indicates a statistically significant difference when compared to the pre-renal group in post hoc pairwise comparison (*P *< 0.05). Click here for file
