# Supplementary figures and images for: Urinary angiotensinogen predicts adverse outcomes among acute kidney injury patients in the intensive care unit
Source: Crit Care. 2013 Apr 15;17(2):R69. doi: 10.1186/cc12612 (PMC3672721; doi:10.1186/cc12612)

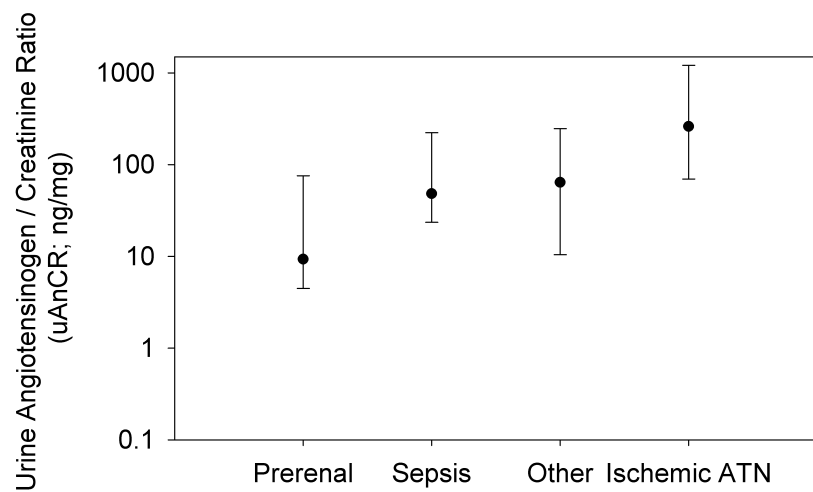

Supplement: Additional file 1 — Urinary angiotensinogen/creatinine ratio (uAnCR) by acute kidney injury (AKI) etiology. Patients who developed AKI in the ICU were grouped by the etiology underlying the AKI. The median (black dot) and interquartile range (error bars) are shown. The * symbol indicates a statistically significant difference when compared to the pre-renal group in post hoc pairwise comparison (P < 0.05). [file cc12612-S1.PDF]
